# Distinguishing True Spillover Trends from Surveillance Bias in Detected Cases

**DOI:** 10.21203/rs.3.rs-10694137/v1

**Published:** 2026-09-02

**Authors:** Kendra Gilbertson, Iris Holmes, Ana Bento, Yining Sun, Caylee Falvo, Daniel Becker, Zulma Rojas-Sereno, Amanda Vicente-Santos, Wanda Markotter, Latiffah Hassan, Marike Geldenhuys, Marinda de Vries, Raina K. Plowright

**Affiliations:** Cornell University; Southern Illinois University; Cornell University College of Veterinary Medicine; Cornell University College of Veterinary Medicine; Cornell University; University of Oklahoma; Cornell University; University of Pretoria; University of Missouri; University of Pretoria; University of Pretoria; Cornell University

## Abstract

Claims that zoonotic spillover is rising globally have shaped research priorities for decades, but this apparent rise can be a statistical artifact of changing detection effort, not a genuine biological signal. Methodological correction alone cannot resolve the ambiguity: closing the gap requires investment in surveillance infrastructure, not modeling. Testing three approaches—generalized linear models, generalized additive models, and double machine learning—against simulated data spanning detection-bias and prevalence conditions, we find trend direction is recoverable under simple bias but not under oscillating or shared-driver detection, and all methods risk false increases when true rates are stable. Applied to 27 years of bovine rabies surveillance in South Africa, independent methods agreed on the declining trend direction but produced magnitude estimates differing more than threefold, showing model disagreement is informative. We translate these findings into reporting guidelines: prioritize direction over magnitude, disclose method reliability, treat cross-model disagreement as signal, and flag high-risk detection regimes.

## Introduction

Zoonotic pathogens are widely discussed as an escalating threat to human health, with spillover rates from animals to humans asserted to be recently or currently increasing^1–4^. Before any policy response to “rising spillover” is justified, it is necessary to know whether that apparent rise reflects real biological change or simply changes in detection and reporting effort — making the surveillance infrastructure question a methodological necessity. These trends were recovered using statistical modeling on historical outbreak data inherently skewed by detection and reporting bias. A growing body of work cautions that apparent increases may partly reflect expanding surveillance and discovery effort rather than genuine change: reporting effort strongly confounds estimated emergence patterns^5^, and the virological data underlying many zoonotic-risk assessments are incomplete and rapidly shifting with ongoing virus discovery, making firm trend claims premature^6^. Despite this complexity, understanding spillover dynamics remains critical to reducing it. Spillover involves the interplay of host population dynamics, environmental change, pathogen evolution, and human behavior, each varying across space and time^7^. Understanding whether and how spillover frequency changes through time is needed to study its putative drivers and the resources required to reduce pandemic threats.

Two fundamental challenges complicate estimating spillover trends. First, datasets are incomplete, and case detection and reporting are shaped by bias that can produce misleading results^8–12^. Most data come from passive surveillance, in which cases are identified by healthcare providers or other institutions and reported to a central data-collection agency^13^; whether a case is reported depends on care-seeking behavior, diagnostic access, provider reporting practices, and data publication^14^. Active surveillance requires public health officials to search for cases of a given disease, theoretically identifying more cases with less bias. However, this effort is costly and rarely sustainable funded^15^, leaving notable gaps in evaluating population health, including correcting for artificially low reporting^16–18^. Acquiring accurate and representative data is the first challenge; followed by methodological decision-making.

The second challenge is choosing among modeling options that correct for bias – such as confounder selection – to recover the true temporal trend. Results are typically sensitive to these choices, making the true trend difficult to recover or validate. Prior work has shown the impact of covariate choice^19–21^, missing-data correction^22^, and other modeling decisions^23^ on health outcomes. It is also not always clear which model output — conditional effects, marginal effects, treatment effects, regression coefficients, or point versus directional estimates — best represents spillover's direction and magnitude. Modelers' choice of central estimate can shape their major conclusions, making this choice critical to our central goal, and these statistical concepts can be difficult to communicate between analysts and the public.

Distinguishing true epidemiological trends from detection artifacts is necessary for public health decision-making, yet few studies have systematically evaluated which approaches recover underlying spillover dynamics from biased data. Previous efforts to estimate true trends while accounting for reporting bias have controlled for infectious disease journal publication counts^2^, or for latitude, GDP, press freedom, internet usage, population size/ density^1^; others sidestepped the problem by selecting pathogens less subject to reporting bias^3^. A key gap in this work is that the proposed adjustments were never tested against simulated data with a known underlying trend, leaving open whether results reflect reality or methodological artifact. Each paper reports increasing rates of spillover events in the relatively recent past (e.g., Jones *et al*. 2008 observed a peak in the 1980s). Here, we step back from the field’s widely accepted claim that global spillover is increasing and test the ability of three different approaches to recover true temporal trends from simulated data with known reporting biases.

We generate data with a known true trend, then ask whether standard statistical methods can recover that truth once realistic surveillance imperfections are layered on top. We simulated four scenarios pairing spillover with a detection process capturing key real-world sources of bias: (1) a linear change in true cases over time, (2) oscillating spillover patterns, (3) detection effort scaling with distance to a test site, and (4) “shared drivers,” where the same factors that influence disease risk also impact detection effort. Each scenario was crossed with three prevalence levels to test the sensitivity of our conclusions. Because simulations have a known ground truth, we can evaluate three models (generalized linear mixed model [GLMM], generalized additive mixed model [GAMM], and double machine learning [DML]) in recovering true trend direction and magnitude across bias conditions. These methods range from traditional regression to modern causal inference and differ in flexibility, confounder handling, and computational complexity, allowing us to test both individual recovery performance and cross-method agreement. High simulation success would support prior claims of changing spillover rates; low success would suggest additional work is needed before concluding that spillover is increasing globally. This design also clarifies which challenges statistical methods can resolve and which require practical solutions like improved data collection. We then apply these approaches, alongside a hierarchical Bayesian model, to a real-world dataset of bovine rabies cases in South Africa, where detection bias is known but true trends are not.

Across our simulations and empirical dataset, we ask: under which bias structures do methods recover trend direction and magnitude, and are failures systematic? These answers help contextualize modeling results for policymakers, particularly regarding uncertainty. We show that methods recover trends reliably under simple bias structures but fail under more realistic oscillating and shared-driver settings. Steady spillover is systematically misclassified as increasing when detection changes over time, a pattern directly relevant to the inference that global spillover is rising.

## Results

### Simulation Direction Recovery

Two main patterns emerged. First, success or failure was driven more by scenario structure than by modeling framework: GLMMs, GAMMs, and DML generally succeeded together (> 50% correct classifications) in linear and distance-based settings and failed together (< 50%) in oscillating and shared-driver settings. Recovery was high when the true spillover trend was steadily increasing/decreasing and detection bias was simple, but deteriorated sharply when trend structure or detection dynamics became more realistic. Second, increasing and decreasing spillover trends showed similar recovery rates, as expected given the inverted symmetry of detection dynamics. The main inferential problem lay less in model class than in the interaction between detection bias and temporal structure.

All but three GLMM models in the steady spillover shared-driver scenario converged. Results from failed models were excluded from the remaining result reporting. Results for the distance-based scenario were very similar to the linear scenario in direction recovery and are reported along with full results for each model in Supplementary Table 5. In our simulations, confidence intervals for estimated annual trends were typically narrow (median width 0.16 for DML, 1.49 for GAMM, and 1.57 for GLMM; interquartile ranges under steady spillover were 0.05–0.22 for DML, 1.08–2.50 for GAMM, and 0.89–2.52 for GLMM), so the ‘steady’ category was not a reflection of highly imprecise estimates. We assessed each method's ability to correctly classify temporal trends as increasing, steady, or decreasing across all remaining simulation scenarios in Fig. 3. Direction recovery was substantially worse in two specific scenarios. First, shared-driver scenarios with increasing/decreasing spillover proved difficult to recover accurately, regardless of the detection trend. Success rates dropped well below 50% for all three methods under these conditions. Shared-driver scenarios with steady spillover performed much better. Second, oscillating scenarios with steady spillover had low success when paired with increasing/decreasing detection. However, when both spillover and detection remained steady in oscillating scenarios, all methods performed well.

In failed shared-driver scenarios where true spillover was increasing/decreasing, models more frequently misclassified the trend as steady rather than identifying the wrong direction. In contrast, when true spillover was steady in shared-driver scenarios, models were generally biased toward an increasing trend (Fig. 4). Oscillating scenarios also exhibited this bias; when the true trend was steady, nearly 100% of misclassified simulations across all models recovered an increasing trend.

The analyses above describe how often each method correctly detected a trend that was truly present. We next asked the reverse question: when a method reported a given trend, how often was that classification actually correct? In oscillating scenarios with decreasing or steady spillover, trend recoveries remained highly accurate for all methods; however, when methods identified an increasing trend, this was correct 60–61% of the time. All incorrect positive trends had a true underlying steady trend. Under shared-driver scenarios designed to simulate realistic detection bias, the reliability of recovered trends differed dramatically across methods. When DML identified a directional trend (increasing or decreasing), that call was correct 55–71% of the time, while a DML report of a steady trend was correct 35% of the time. GLMMs and GAMMs were less reliable: a reported directional trend was correct 35–38% of the time and a reported steady trend was correct 33–34% of the time.

### Systematic Positive Bias Under Steady True Trends

When the true trend was steady, all three methods showed a directional bias toward false positives, but the strength and structure of this bias differed sharply across scenario types. The bias was most pronounced under oscillating scenarios: with steady spillover and changing detection, GLMMs misclassified the trend as increasing in 92% (increasing detection) and 99% (decreasing detection) of scenarios, with similar rates for GAMMs (94% and 97%) and DML (both 100%), and no misclassifications in the decreasing direction (Fig. 4). Under shared-drivers the same asymmetry was present in relative terms (GLMM increasing detection: misclassified as increasing 4% vs. 1% as decreasing; GLMM decreasing detection: misclassified as increasing 12% vs. 0% as decreasing; see Fig. 4 for GAMM and DML) but represented only 7% (GLMM), 21% (GAMM), and 5% (DML) of replicates in absolute terms. The dominant failure mode under shared-drivers was not directional bias but insensitivity: methods overwhelmingly defaulted to “steady” (~ 91% of increasing or decreasing trends recovered a steady trend for GLMM) and lacked power to identify any direction. Linear and distance-based scenarios showed minimal bias in either direction (≤ 7% of replicates misclassified under steady spillover). These two failure modes (directional bias under oscillating detection and insensitivity under shared-drivers) have distinct implications for fielded analyses: oscillating-detection systems risk affirmatively producing spurious increasing trends, while shared-driver systems risk failing to detect real change at all.

### Simulation Point Estimate Recovery

We next evaluated point trend recovery by assessing whether 95% confidence intervals from GLMMs and GAMMs contained the true annual percent change (Fig. 5). As with direction recovery, GLMMs and GAMMs performed similarly, though GLMMs achieved marginally higher success rates in shared-driver scenarios with steady spillover. Failure rates were substantially higher for point estimate recovery compared to direction recovery across all scenarios. Several scenario combinations exhibited near or complete success in direction classification but near-total failure in point estimate capture. Specifically, oscillating scenarios with increasing spillover and either increasing or decreasing detection, showed 100% direction success but 0% point estimate success. The same pattern holds for oscillating scenarios with decreasing spillover paired with changing detection. This discrepancy indicates that while methods correctly identified the sign of temporal trends, they systematically over- or under-estimated the magnitude of change. Consistent with direction recovery results, oscillating and shared-driver scenarios had worse point estimate recovery than linear scenarios.

### Prevalence Level Sensitivity

Under the medium prevalence scenarios, all GAMM models converged across all scenarios and simulations. GLMM convergence failures occurred at low rates across scenarios: linear trend (2.2%), distance-biased detection (2.4%), oscillating (1.7%), and shared-drivers (1.4%). Confidence intervals for estimated annual trends from the parametric models were typically narrow and slightly wider than at high prevalence (median width 3.66 for GLMM and 3.81 for GAMM, steady-spillover IQRs 2.10–5.75 and 2.51–5.80). DML intervals, by contrast, narrowed relative to high prevalence (median 0.05, IQR 0.02–0.08), the result of regularization-driven contraction. Directional and point recovery for GLMM and GAMM were largely insensitive to the reduction from high to medium prevalence, with recovery of genuine increasing/decreasing spillover trends essentially unchanged (Supplementary Table 6). There was a substantial increase in success for GLMM and GAMMs in oscillating scenarios with steady spillover under increasing/decreasing detection (e.g. GLMM 8%→72% under increasing detection). DML performance deteriorated substantially to 50% or below under increasing/decreasing true trend scenarios with increasing/decreasing detection bias. Under point success, GLMM and GAMM results remained largely stable (Supplementary Fig. 3). A notable exception again occurred in the oscillating trend with steady spillover and increasing/decreasing detection, where directional success for both models shifted from majority failure to majority success. The asymmetry toward false positives over false negatives in the oscillating scenario was preserved at this prevalence for all three models with misclassification of steady spillover remaining overwhelmingly in the positive direction. The false-positive rate itself declined relative to high prevalence, while the shared-driver scenario showed a reduction in asymmetry (Supplementary Fig. 4).

Under the low prevalence scenarios, all GAMM models converged across all scenarios and simulations. GLMM convergence failures occurred at low rates across scenarios: linear trend (4.2%), distance-biased detection (4.0%), oscillating (3.0%), and shared-driver (2.3%). For the parametric models, confidence intervals widened markedly relative to medium and high prevalence (median width 11.81 for GAMM, and 11.67 for GLMM; steady-spillover IQRs 8.41–16.87 for GAMM, and 9.09–16.97 for GLMM), whereas DML intervals narrowed further (median 0.02, IQR 0.01–0.03). For GLMM and GAMM, widening CIs reflect a loss of statistical information, while their point estimates stay essentially the same. DMĽs intervals narrowed even as its ability to recover trend direction collapsed. This narrowing does not reflect greater precision, it reflects a known property of the underlying machine-learning method, where regularization – a process that penalises extreme estimates to prevent overfitting – pulls weak or noise signals toward zero, producing a falsely confident null trend. At this prevalence the robustness seen at medium prevalence broke down: directional recovering of increasing and decreasing spillover fell sharply for all models (e.g. linear increasing spillover under increasing detection: GLMM 100%→43%, GAMM 100%→51%, DML 40%→7%; Supplementary Table 7), with the same pattern for decreasing detection and decreasing spillover. For the parametric models this reflected standard-error inflation rather than any change in the estimates themselves. The recovered year coefficients remained close to their high-prevalence values and to the true trend while the surrounding intervals widened past zero, so misclassified replicates were almost exclusively reassigned to steady rather than to the opposite direction, indicating false negatives driven by insufficient power (Supplementary Fig. 6). In contrast, oscillating scenarios with steady spillover under increasing/decreasing detection showed further improvement, as the spurious detection trend fell below the significance threshold. The false-positive bias under steady oscillating spillover persisted across all models (Supplementary Fig. 5).

### Bovine Rabies Case Study

All four methods converged successfully and passed model diagnostics when applied to the South African bovine rabies surveillance data. The GLMM estimated a temporal trend of −2.74% change in bovine rabies cases per year (95% CI: −3.56% to −1.92%) (Fig. 6). The GAMM estimated a steeper decline of −9.64% per year (95% CI: −10.39% to −8.87%). The P-spline on distance to diagnostic laboratory had an effective degrees of freedom of [edf = 23.00], indicating approximately nonlinear structure in the detection relationship. DML estimated an annual decrease of 0.003 bovine rabies cases per district (95% CI: −0.005 to −0.003, and the hierarchical N-mixture model estimated − 2.5% per year (95% credible interval: −3.38 to −1.60). This comparison shows that agreement on trend direction does not imply agreement on scale or estimand.

## Discussion

Our central finding is that both standard and state of the art statistical adjustments to account for detection and reporting bias of cases of zoonotic spillover do not reliably recover spillover trends from biased surveillance data. Across three modeling frameworks, the most consequential failure pattern was a directional bias toward spurious positives when the true trend was steady, especially in the oscillating scenario. Our bovine rabies case study reinforces this point by showing that even when methods agree on trend direction, they can still disagree substantially on magnitude.

This pattern relates directly to the empirical claims that motivated our study. Previous studies have controlled for correlates of detection assuming a linear change in spillover rates^1,2^. Covariates explicitly addressing the configuration that produces the largest bias in our simulations, oscillating detection paired with steady or near-steady true incidence, or detection trends that share environmental and infrastructural drivers with spillover risk itself are rarely found in multi-pathogen analyses of spillover. Meadows et al. (2023) sidestepped the adjustment problem by restricting attention to pathogens thought to be less susceptible to reporting bias; this strategy assumes any residual bias is too small to systematically distort trends, an assumption complicated by our finding that the errors introduced by residual detection bias often fail in one direction, so even small departures from perfect detection can accumulate into a spurious upward trend. None of these efforts validated their bias-correction approach against a known ground truth. Their reported increases in spillover frequency may therefore be at least partially driven by the directional artifact we document here, rather than reflecting genuine global escalation. We are not claiming the rising trends in these and similar papers are entirely artifactual (our simulations do not rule out an increasing trend in pathogen spillover or pathogen outbreaks), but we are claiming that the methods used cannot, on their own, distinguish the two possibilities.

While trend recovery was difficult, point recovery (the proportion of simulations where the 95% confidence interval for the estimated trend contained the true percent change per year) had additional limitations. Even in cases where models recovered the correct trend direction, confidence intervals often failed to capture the true magnitude of the underlying trend. We therefore recommend against relying on point estimate recovery for communicating spillover trends in all but the simplest or most well-documented surveillance systems with minimal bias. For example, reportable high-consequence pathogens such as human cases of rabies are likely to be identified in surveillance data. We recommend that researchers focus on establishing trend direction with appropriate uncertainty rather than presenting precise magnitude estimates that convey false confidence.

When approaching this alternative perspective—how often is the recovered trend true—we found very low reliability across our shared-driver scenarios. Both GAMMs and GLMMs were correct in 33–38% of the simulations. DML was notably more reliable when it recovered either a decreasing or increasing trend, though its ability to correctly identify a steady trend was still low. The distinction between the two outcomes (whether the true trend is recovered vs. whether the recovered trend is true) reflects alternative statistical perspectives as well as two different questions in public health surveillance. The probability a method recovers a true trend addresses whether our surveillance system can capture genuine shifts in disease dynamics. However, the probability that a recovered trend is true addresses the question public health decision-makers are more likely to face: given the results of the analysis, should we act on them? From this perspective, even imperfect methods can still provide utility if their reliability is known. The 55–71% accuracy of DML when identifying directional trends under shared-drivers is imperfect, but it still provides a defensible basis for action, particularly when weighed against alternative statistical approaches and the practical limitations of epidemiological research. However, it is important to note that DML still had a failure rate > 50% when the recovered trend was steady.

Poor trend recovery performance in steady oscillating scenarios under changing detection is concerning given the number of zoonotic pathogens that exhibit seasonal patterns^24–26^. This pattern likely reflects the models’ difficulty in distinguishing short-term seasonal variation from the underlying long-term trend, an issue that may become more pressing as climate change shifts or amplifies seasonal patterns^27^. The inferential limitations we have identified here extend to forward-looking risk assessments as well as historical patterns. This is concerning for both vector-borne pathogens and other zoonoses with strong seasonal dynamics. Vector-borne diseases account for over 17% of infectious diseases and disproportionately affect poorer populations^24^. The difficulty of quantifying the temporal trends of such diseases may be compounded by the relatively limited resources available to study them comprehensively over time. However, while the proportion of simulations in which the true trend direction was correctly recovered was low in certain oscillating scenarios, the proportion of calls that were made being correct was higher (60–100%).

The medium and low prevalence simulations show that recovery is governed by how detection power scales with prevalence, with differing causes of failure by model class. In plain terms: as prevalence fell, the models' estimates of the trend barely changed, but the uncertainty around those estimates grew — so trends that had looked significant at high prevalence simply became statistically indistinguishable from “no trend” as data got sparser. Less data made the models look paradoxically more accurate in the oscillating scenario because it was the spurious signal — not the true one — that vanished first. For the parametric models, this happens because each observed count is a binomial thinning of the true count, so detectable signal scales with the number of true cases available. The spurious trend in the oscillating scenario and the true trend in the linear scenario stayed close to their high-prevalence values as prevalence fell — it was the widening interval, not a change in the estimate itself, that drove reclassification to “steady.” This resolves the apparent paradox that less signal yields greater success in the oscillating scenario: the signal lost there is the detection artifact, and as the uniformly inflating interval overtakes the small spurious coefficient, the true steady process is correctly recovered. The same inflation is harmful where the trend is real: because the interval inflates uniformly while coefficients hold their magnitude, the small artifactual coefficient loses significance first (by medium prevalence) and the larger genuine coefficients later (by low prevalence), so the two phenomena are one mechanism resolved in order of effect size rather than two separate behaviors. The dominant error type accordingly shifts with prevalence — over-detection of trends, including artifactual ones, at high prevalence (false positives), and under-detection, including of real ones, at low prevalence (false negatives). DMĽs failure took a different form entirely. Rather than inflating its interval around a stable estimate, it shrank both the point estimate and its standard error toward zero, producing falsely precise null intervals. This contraction was already apparent at medium prevalence but became pathological only at low prevalence, once the shrinking coefficient settled around zero. This explains why DMĽs interval widths moved opposite to the parametric models and did not improve to the extent of the parametric models at medium prevalence. This raises a concern we return to below: sparse data from limited detection capacity may make this failure mode systematically worse in low income areas.

When true spillover rates were steady, our models overwhelmingly made errors toward false positives at every prevalence level, even while the false-positive rate itself fell. This reinforces our conclusion that care should be taken before concluding that spillover patterns are increasing over time, particularly where prevalence is low. Because we have only sampled three calibration points along a continuous prevalence gradient, and because prevalence demonstrably moderates performance non-uniformly across scenarios and bias structures, we cannot yet establish whether our headline findings hold across outbreak or epidemic conditions. Pending such a sweep, the conclusions reported here, including the consistent positive bias under steady true trends, most notable in oscillating scenarios, should be read as conditional on the tested range rather than as universal properties of the methods.

Beyond selecting analytical methods, researchers must carefully consider how to communicate results. Our simulations demonstrate how different analytical approaches and modeling decisions can produce different results. The divergent magnitude of recovered trends between GLMM and N-mixture model and GAMM in our bovine rabies results clearly demonstrates the impact of these modeling choices on the results. The GLMM and GAMMs differ in count distribution (Poisson vs negative binomial) and functional form (a smooth versus linear term for distance to the laboratory in the count component), likely both contributing to the difference in magnitude of the recovered trend. The choice of communication also affects how findings are interpreted and acted upon^29,30^. As researchers, it is our responsibility to select metrics that accurately convey the magnitude and uncertainty of detected changes and is most interpretable for the intended audience. When working with stakeholders who will use these analyses for decision-making, involving them early in the analytical planning process ensures that results address their actual information needs. We consolidate these implications into concrete recommendations for two audiences (Box 1).

Given the methodological limitation we demonstrated, we believe that addressing the spillover question requires prioritizing data quality and completeness as well as sound analysis. Our simulations showed that no statistical method can be relied on to extract true trends from incomplete and/or biased detection data, even in relatively simple scenarios, when issues such as shared-drivers and detection bias are present. Specifically, gaps in surveillance and gaps in our understanding of surveillance effort are key challenges. For passive surveillance, reporting is often incomplete and depends on healthcare cost, access to healthcare and access to diagnostic technology as well as healthcare providers’ knowledge of central databases, ability to access them, and willingness to do so^9,31,32^. Before selecting analytical approaches, researchers should consider whether their datasets suffer from similar limitations, engaging subject matter experts and epidemiologists to understand system dynamics through tools like directed acyclic graphs (DAGs) that make causal assumptions explicit^33^. For existing passive surveillance datasets, targeted supplementary data collection could substantially improve inference: structured expert elicitation could quantify how detection probabilities have changed over time, while validation studies comparing detected cases against independent sampling could empirically estimate ascertainment rates.

More fundamentally, zoonotic disease surveillance requires sustained investment in active surveillance systems, particularly in historically neglected regions where spillover risk may be highest but detection infrastructure weakest^5^. Where resource constraints preclude active surveillance, passive systems should minimally report surveillance effort metadata alongside case counts, including the total number of tests performed, healthcare-seeking patterns, laboratory capacity changes, and other indicators that allow analysts to distinguish true epidemiological trends from artifacts of detection to the best of their ability. We emphasize the equity implications of this asymmetry. In our modeling scenarios, we found that detection bias tends to be most pronounced in regions where surveillance infrastructure is weakest, which are also the regions where spillover risk is hypothesized to be highest. The methodological pattern we documented (that statistical methods systematically recover spurious increasing trends from steady true trends when detection probability is changing) therefore disproportionately mislocalizes spillover risk in low- and middle-income settings, both by inflating apparent risk in places where surveillance is improving and by understating risk where surveillance is contracting. Investment in surveillance capacity in historically under-resourced regions is consequently not only an epidemiological priority but a methodological one: it is the only durable solution to a class of inferential failures that no analytic adjustment can fully resolve.

Our study has several limitations that suggest directions for future research. While our simulations incorporated common bias patterns, real-world surveillance data will inevitably be more complex. The bovine rabies analysis was intended not as a definitive assessment of rabies dynamics in South Africa, but rather as a real-world demonstration of how different analytical approaches behave when applied to imperfect passive surveillance data and results may differ substantially for pathogens with different transmission dynamics or environmental systems. Different variable specifications for each model type is likely to change the results we observed in our simulations. Because the N-mixture model could only be stabilized in simulation through strongly informative priors centered on the true values, we cannot characterize its trend-recovery performance under the same prior-agnostic conditions applied to the other methods; this prior dependence, a known consequence of the weak identifiability of abundance and detection from sparse count data^34^, is itself a limitation on the model's applicability to real surveillance datasets where the truth is unknown. Our three prevalence regimes (high, medium, and low) bracket a plausible range for routine zoonotic surveillance, but a continuous prevalence sweep would more fully characterize how method performance scales with signal strength, and outbreak-scale prevalence may behave differently still.

Our simulations also assume that sites are conditionally independent given the random intercept; real surveillance data typically exhibit residual spatial autocorrelation that can inflate Type I error rates and is not represented here, and we did not test method performance under explicit spatial covariance structures (e.g., conditional autoregressive or Matérn-correlated terms). Finally, our simulations treat case counts as the observation unit, but many zoonotic surveillance systems instead report incidence rates with population denominators that vary across space and time, which can introduce a separate set of biases not addressed here. We also note that our simulations span a ten-year window, chosen to match the temporal granularity of most routine surveillance datasets; many influential claims of rising spillover (e.g., Jones et al. 2008) span three to four decades, and whether the bias patterns we identify amplify, attenuate, or qualitatively change over longer time horizons is an open question that warrants explicit investigation.

Despite these limitations, our findings suggest an updated re-examination of reported global trends in zoonotic spillover using alternative methodologies. Applying DML, hierarchical models, or related approaches to previously analyzed datasets could either corroborate existing conclusions about increasing spillover frequency, or suggest that the trend is not as concrete and further examination is necessary.

Statistical models alone cannot solve the spillover trend problem when surveillance data are fundamentally incomplete or biased. We urge particular caution when confronted with overconfident conclusions about spillover trends, especially when expected rates of change are small and may be easily obscured by fluctuations in surveillance intensity, diagnostic practices, or reporting infrastructure that pose additional challenges for statistical adjustment. Our work identifies serious methodological challenges, but also provides a pathway for future exploration as we aim for constructive skepticism rather than nihilism about our ability to understand global spillover and other temporal trends subject to detection bias. These questions remain inherently difficult, and honest uncertainty may serve public health goals better than false precision.

## Methods

We addressed the study questions in two parts. First, we developed simulation scenarios that mimic common biases in spillover surveillance data, from simple changes in detection probability over time to more complex issues of shared-drivers. We compared three statistical approaches representing a spectrum of modeling philosophies: generalized linear mixed models (GLMMs), generalized additive mixed models (GAMMs), and double machine learning (DML) at three levels of prevalence. Second, we applied these methods, along with a hierarchical N-mixture model, to bovine rabies surveillance data from South Africa as a real-world case study where some detection biases are known to exist but true trends are unknown. Analyses were carried out using R 4.4.2 (R Core Team, 2024). A diagram of the full methods process can be found in Fig. 1.

### Simulation Scenarios

#### Scenario Types.

We designed four simulation scenarios representing common biases in spillover data, ordered from simple to complex:

1. **Linear**: As the base scenario, both true spillover rates and detection probability changed gradually and consistently over time. This represents situations where disease risk and surveillance effort both follow steady trends, such as a disease becoming more common as diagnostic capacity improves. This is expected to be the easiest trend to recover and serves as a reference for more complex scenarios.
2. **Oscillating**: True spillover rates exhibited seasonal fluctuations superimposed on an underlying linear trend, mimicking zoonotic diseases with a seasonal transmission cycle. Detection probability changed linearly over time without seasonal variation, creating temporal mismatches between disease risk and surveillance effort.
3. **Distance from testing site**: True spillover rates changed linearly over time, but detection probability varied spatially rather than temporally. Each site was randomly assigned a distance to a theoretical diagnostic testing center; sites farther from the center had lower detection probabilities throughout the study period. This represents geographic disparities in surveillance.
4. **Shared-drivers**: True cases followed a linear trend with seasonal variation, while detection probability responded to the same underlying environmental and infrastructural factors. This scenario represents a common challenge in epidemiological surveillance where the same factors influencing disease risk can also increase or decrease detection effort. For example, areas with higher rainfall might experience both increased vector populations (raising true spillover risk) and improved infrastructure funding (raising detection probability).

While we acknowledge that realistically all surveillance data would have several of these factors (i.e. spatial structure and autocorrelation and shared-drivers), our goal was to test the impact of each factor individually.

#### Simulation-Generation Process.

In brief, each of our simulated datasets have two layers: the true number of spillover events that occurred, and the subset of those events that surveillance actually captured. We generated these separately and then combined them in a third model to obtain the number of observed cases for the scenario. For each simulation, we first generated true primary spillover cases from animal to human using a Poisson regression model with time-varying environmental covariates (full rate calculations in Appendix A). The rate parameter for the expected number of events was determined by whether the underlying trend was increasing, decreasing, or stable. Specifically, we modeled rate on the log scale as a linear function of the time-varying covariates, where the intercept and slope coefficients were tuned to produce realized monthly case counts matching the prevalence regimes described below. Full simulation specification and parameter values are provided in Supplementary Note 1. This produced monthly case counts at each of multiple study sites, representing the unobserved reality of spillover occurrence. We then modeled the detection process separately using logistic regression, which generated site- and time-specific detection probabilities ranging from 0 to 1. We combined these components by drawing from a binomial distribution: for each site and month, the number of detected cases was determined by the number of true cases and the probability of detection. This created realistic datasets where observed cases depend on both underlying disease dynamics and surveillance intensity.

#### Covariate Structure.

The linear, oscillating, and distance scenarios were controlled by two time-varying features: temperature (representing any environmental variable affecting spillover risk) determined the number of true cases, while surveillance infrastructure determined detection probability. To keep these scenarios simple, both temperature and surveillance infrastructure were highly correlated with year to generate the changes over time. The shared-driver scenario used a more complex combination of covariates, better representing realistic situations where multiple factors jointly influence both disease occurrence and detection. In this case, spillover rate was determined by elevation, temperature, wildlife density, and resource availability, while detection probability was determined by temperature, wildlife density, surveillance infrastructure, and resource availability.

#### Factorial Design.

For each of the four scenario types described above, we tested nine combinations of true spillover trends (i.e., increasing, steady, or decreasing) and detection probability trends (i.e., increasing, steady, or decreasing). With the 200 stochastic replicates per cell, this yielded 1,800 simulations per scenario type (9 cells × 200 replicates) and 7,200 simulations across the four scenario types in the primary analysis for each level of prevalence. This three-by-three factorial design allowed us to assess whether statistical methods could distinguish genuine changes in spillover rates from artifacts of changing surveillance effort. Specific parameter values were chosen to illustrate plausible magnitudes across a ten-year period. Detection probabilities were consistent across all scenario types and prevalence levels: steady detection was maintained at approximately 0.55 (detecting about half of all cases); increasing detection rose from 0.35 to 0.90; decreasing detection declined from 0.90 to 0.40.

Because simulation performance may depend on signal strength, we evaluated three spillover prevalence regimes, high, medium, and low, while keeping the bias structure and study design fixed. Under the high-prevalence regime, true spillover rates ranged from approximately 6 to 10 cases per site per month in increasing and decreasing scenarios, with steady scenarios maintaining approximately 6 cases per site per month. The medium-prevalence regime gave expected true case rates roughly one order of magnitude lower than the high-prevalence regime (midpoint approximately 0.9 cases per site-month), and the low-prevalence below that (midpoint approximately 0.1 cases per site-month). The parameter values and analytical derivations underlying these expected counts are provided in Appendix A.

The low-prevalence regime is relevant to emerging zoonoses and novel pathogens where most site-months have zero cases even in the absence of detection bias. At very low rates, Poisson-based regression methods can have poorly calibrated confidence intervals due to the slow approach to asymptotic normality, and the structural distinction between true zeros and detection failures becomes harder to identify. Method performance under these conditions is an important practical question. Results from the high-prevalence group are presented in the main text; medium and low results are reported in the sensitivity analysis and supplement. Outbreak or epidemic-scale prevalence, in which monthly counts are an order of magnitude higher than our base scenario, were not explored and may yield qualitatively different performance profiles given the higher signal-to-noise ratio.

### Modeling Approaches

#### Study Design.

Each simulation scenario included 70 sites sampled once per month over a 10-year period, generating 8,400 total observations per simulation. We ran 200 independent realizations of each scenario to assess method performance under random variation. Pilot runs indicated that results stabilized before approximately 100 replicates; we doubled this to 200 to provide additional precision. At this sample size, the Monte Carlo standard error for any estimated proportion is at most 3.5 percentage points (at p = 0.50) and falls below 2.2 percentage points for proportions above 0.90 or below 0.10, ensuring that our reported direction-recovery rates are not dominated by simulation noise. We then evaluated our three statistical approaches for estimating spillover trends from surveillance data.

#### GLMMs.

GLMMs relate observed spillover counts to linear change in explanatory variables, estimating the relative importance of each linear predictor in explaining variation in the data. We fit negative binomial models to account for overdispersion common in count data, with random intercepts for site to reflect repeated sampling at the same locations. Each model included a variable for the calendar year to estimate the temporal trend in observed cases. One of the key issues we bring to the reader’s attention is the decision of which covariates to include to control for detection bias:

- **Separate-driver scenarios** (linear, oscillating, distance): We included only the surveillance infrastructure variable used to generate detection probability in the simulations while excluding the temperature variable that determined true spillover rates. We included a quadratic polynomial term on surveillance infrastructure using the *glmmTMB* (*v1.1.11*; Brooks *et al*. 2017) package. This approach attempted to isolate the effect of time on true cases by controlling for known detection drivers.
- **Shared-driver scenario**: We included all variables that influenced change in detection probability over time in the simulations with quadratic polynomial terms. However, this creates a methodological issue: by controlling for variables that affect both detection and true spillover risk, we simultaneously remove both the detection bias we aim to eliminate and part of the causal pathway between time and true cases we attempt to recover. This represents a common dilemma in epidemiological analysis when risk factors and surveillance drivers overlap. Shared-drivers may also cause opposite reactions. In many real surveillance systems, the same factors that elevate spillover risk also reduce detection capacity. Areas affected by ecological degradation, rural poverty, or weak public health infrastructure may be more vulnerable to spillover while simultaneously having reduced surveillance coverage, diagnostic access, and reporting completeness (cites). Our factorial design across all combinations of true-trend and detection-trend captures both regimes, including cases in which the two move in opposite directions.

All scenarios were fit using a negative binomial distribution given the overdispersion of the response data. We acknowledge that the strong correlation between year and surveillance infrastructure, necessary to generate simple temporal trends in our simulations, introduces multicollinearity that is not ideal for modeling. However, biological and epidemiological variables often co-vary with time, making our simulations a useful test of method performance under realistic conditions. The recovered temporal trend was estimated by converting the year coefficient from a log rate ratio to percent change per year as (*exp*(*β*)–1)*100. Convergence was assessed via successful optimizer exit (convergence code = 0), a positive-definite Hessian, finite standard errors for all fixed-effect coefficients, and an estimated dispersion parameter greater than 0.01.

#### GAMMs.

GAMMs extend GLMMs by allowing nonlinear relationships between predictors and spillover counts through the use of smooth functions. This flexibility is valuable when the relationship between predictor and outcome is unknown or suspected to be nonlinear, as in many ecological relationships. GAMMs followed the same structure as GLMMs, including the same covariates, distribution, and random effects. The primary difference is that GAMMs can capture nonlinear temporal trends and nonlinear effects of detection covariates without requiring the analyst to specify the form of the relationship. In the linear, oscillating, and distance simulations surveillance infrastructure and site were smoothed using the default thin plate spline in the *mgcv* (*v1.9–3*; Wood 2017) package. We used the same smoothing function in the shared-driver scenario on surveillance infrastructure and site, as well as wildlife density and resource availability which were not present in the other scenario types. We calculated the effect of year using the same approach as GLMMs, giving percent change per year on the multiplicative scale. Year was a parametric (linear) term in the GAMM rather than being smoothed, so that the year coefficient retained the same interpretation as in the GLMM and could be converted to an annual percent change in the same way. This is a deliberate design choice: the GAMM’s added flexibility was used to capture potentially nonlinear effects of the detection-related covariates, while the trend term itself was constrained to a constant annual rate to preserve a single, directly comparable estimate of temporal change across methods. As a consequence, any nonlinearity in the underlying trend (for example, in the oscillating scenarios) is absorbed into residual variance and the parametric covariate effects rather than into the year term. Convergence was assessed by successful completion of the PIRLS optimizer, finite standard errors for all model terms, and a finite GCV/REML score.

#### DML.

DML is a fundamentally different modeling approach that explicitly separates the estimation of detection processes from the estimation of true spillover trends. The method, using the *grf* (*v2.4.0*; Tibshirani *et al*. 2026) package, contains three phases:

1. **Detection model**: Estimates how observation effort varies across time and space.
2. **Outcome model**: Estimates the expected number of detected cases across time and space.
3. **Estimation**: Combine these two estimates to account for detection bias and predict the true number of cases across time and space.

Each phase uses random forests (RF), a machine learning algorithm that groups observations with similar predictor values and outcomes using multiple, random subsets of data. Like GAMMs, RFs can capture nonlinear relationships without pre-specifying functional forms. Because DML explicitly models the detection process separately, all covariates related to either detection effort or static environmental heterogeneity can be included in both the detection and outcome models, avoiding the variable assignment issues we encountered with GLMMs in shared-driver scenarios. The DML approach also avoids the need to include a dummy “time” covariate to estimate the trend in cases. This makes DML appealing for applications with shared-drivers, though the decision of which variables to include can quickly become complex, and its implementation is more advanced than GLMMs or GAMMs. DML is more computationally intensive than regression approaches and requires cross-validation to prevent overfitting. We followed the implementation described in Fink et al. (2023), which provides detailed guidance on model structure. Additionally, the DML approach assumes that all spatial and temporal variation in detection effort and all spatial variation in underlying spillover rates is accounted for by the covariates provided to the model, an assumption that is unlikely to be fulfilled in practice^35^.

DML directly estimates the average treatment effect (ATE) as its primary output. Conceptually, DML isolates the change in cases over time that remains after mathematically removing the portion attributable to detection-related variables — analogous to residualizing out a confounder before estimating a trend. DML reports this residual trend, the ATE, as an absolute change in case counts per year (additive scale) rather than percent change (multiplicative scale). This means DML trend estimates cannot be directly compared in magnitude to GLMM/GAMM estimates or to the true trend, although the direction (increasing, steady, or decreasing) remains comparable across methods.

#### True Trend Estimation.

To establish a benchmark for evaluating model performance, we fit Poisson regression models to the true (unobserved) spillover counts generated in each simulation using calendar year as the sole predictor. The exponentiated year coefficient was then converted to a percent change per year as (exp(β) − 1)×100, yielding the annual rate of change in true underlying incidence. This benchmark reflects the total trend in true cases over time — including any changes driven indirectly through temperature — rather than isolating the effect of year alone. It served as the reference value against which trend estimates recovered by each candidate method were evaluated.

For simulations in which the true spillover trend was steady (neutral), we assessed whether misclassification errors were systematically biased toward false positive (incorrectly classified as increasing) versus false negative (incorrectly classified as decreasing) predictions. For each combination of method, scenario family, and detection pattern with at least 10 total errors, we tested whether the proportion of false positive errors differed significantly from 0.5 using a two-sided binomial test, with Bonferroni correction applied across all comparisons (*broom* package).

#### Performance Evaluation.

We assessed each method's ability to recover true spillover trends using directional accuracy and precision. For directional accuracy, we classified each trend estimate (true trend, GLMM, GAMM, and DML) as increasing (95% confidence interval entirely above zero), steady (95% CI overlapping zero), or decreasing (95% CI entirely below zero). A method was considered successful if its classified trend direction matched the true trend direction. We evaluated how often a method’s classification was correct when it detected a trend, and how often a method correctly detected a trend that was truly present. For GLMMs and GAMMs only (due to scale differences with DML), we calculated the proportion of simulations where the 95% confidence interval for the estimated trend contained the true percent change per year. This precision metric evaluates whether methods capture the true parameter value within their uncertainty interval. Together, these metrics assess whether each method detects the correct pattern (i.e., directional accuracy) and whether they appropriately quantify uncertainty around that pattern (i.e., quantitative precision).

### Applications to Bovine Rabies Surveillance Data

#### Case Study Description.

To evaluate method performance on real-world surveillance data, we applied the three modeling approaches, as well as a Bayesian hierarchical model, to bovine rabies cases from South Africa spanning 1993 to 2019 (Fig. 2). This dataset is a useful test case for several reasons. First, rabies virus cases in cattle represent spillover events rather than transmission chains, as cow-to-cow transmission is negligible^36^. This means temporal trends in detected cases should reflect either changes in spillover from reservoir hosts or changes in surveillance effort, not an increase in transmission within the species, making it directly analogous to our simulation scenarios. Second, expert opinion suggests that distance from diagnostic testing facilities and associated logistical challenges affect the likelihood that cases are identified and reported, providing a known source of detection bias similar to our distance-based simulation scenario (the assumed causal structure of the bovine rabies system is shown in Supplementary Fig. 1). Finally, the dataset exhibits the types of shared-drivers common in real epidemiological systems; factors like canine, jackal and mongoose rabies prevalence likely influence both the true risk of bovine spillover, through increased exposure, and the probability of detection, through heightened awareness and surveillance effort^37^. Due to these factors, the rabies case study most closely resembles our shared-driver simulation.

The dataset contains information on monthly rabies outbreaks across 210 districts, small political units within South African provinces. Cases in cattle, domestic dogs, mongooses, and jackals are most commonly recorded, though other host species are occasionally documented. Veterinary staff, who are responsible for reporting rabies cases to the South African federal government, typically cover multiple districts, with several operating within each province. Reported cases are followed up and diagnosed through the fluorescent antibody test on collected brain tissue, though diagnostic confirmation is further constrained with only two reference laboratories nationally performing rabies diagnostics. Both the tendency of citizens to report cases and the capacity of veterinary services to follow up vary across space and time, creating heterogeneous detection probabilities that appear in many disease datasets.

The case data had high zero-inflation, with many district–months recording no detected cases. In addition to case counts, the dataset included distance from each district to the nearest diagnostic laboratory, canine and mongoose rabies cases in the same district, and we obtained monthly precipitation and temperature (relevant to spillover rates) as well as human population density. These predictors showed low correlation (< 0.27) with each other or year (Supplementary Fig. 2). Variance inflation factors were below 1.03 for all predictors (Supplementary Table 4), indicating negligible multicollinearity.

We hypothesized that canine rabies cases, mongoose rabies cases, human population density, and distance to testing facilities influenced case detection probability. Detection effort is not uniform across species: bovine carcasses represent a direct economic loss and are disproportionately likely to be recovered and submitted for testing, whereas wildlife rabies deaths often occur in remote areas without carcass recovery, and free-ranging domestic dogs are similarly under-ascertained despite frequent human contact. This asymmetry means that bovine surveillance data are shaped not only by the biological process generating spillover but by a detection process systematically biased toward economically valuable, easily observed hosts. We also expected canine and mongoose cases to causally affect the number of true bovine spillover events, making them shared-drivers that influence both the biological process and the observation process.

#### GLMM Application.

We fit a zero-inflated negative binomial (ZINB) GLMM using maximum likelihood in the *glmmTMB* package to accommodate both the excess zeros and overdispersion in the bovine case data. The count component of the model included distance to diagnostic laboratory and human population density as covariates to control for spatial variation in detection probability, along with a continuous year variable to estimate temporal trends. The zero-inflation component included canine rabies cases to model the probability of observing zero outbreaks versus any positive count (mongoose cases were dropped to improve model fit). A random intercept for the district accounted for repeated observations at the same locations over time. This structure allows the model to distinguish “true zeros” (i.e., months where no spillover occurred) from “false zeros” (i.e., months where spillover occurred but was not detected), while controlling for known detection drivers in the trend estimation. Like the simulations, the annual trend was recovered by converting the year coefficient to a percent.

#### GAMM Application.

The GAMM used a zero-inflated Poisson (ZIP) distribution using the *gamlss* (*v5.4–22*; Rigby and Stasinopoulos 2005) package as the base model. The count component controlled for distance to laboratory and human population density but applied a penalized beta spline (P-spline) to distance to allow for potential nonlinear relationships between proximity to diagnostic facilities and detection probability. The zero-inflation component included canine rabies cases, and the model included random intercepts for the district and was fit using the Rigby and Stasinopoulos (RS) algorithm. This specification mirrors the GLMM structure but provides additional flexibility to capture complex spatial patterns in surveillance coverage. The annual trend was returned using the same method as the GLMM application.

In preliminary analyses, Poisson models for the rabies case counts showed clear overdispersion relative to the mean, so we adopted a zero-inflated negative binomial specification for the GLMM. We attempted to fit an analogous zero-inflated negative binomial GAMM using the ZINBI family in *gamlss*, but these models did not converge reliably. As a result, the GAMM uses a zero-inflated Poisson distribution, whereas the GLMM uses a zero-inflated negative binomial, so the two models differ both in count distribution (Poisson vs negative binomial) and functional form (a smooth versus linear term for distance to the laboratory in the count component). Accordingly, differences in their estimated trends may reflect either or both modeling differences.

#### DML Application.

The DML implementation required careful specification of three model components. The treatment model (estimating change in detection probability through time) used site-level baseline means for canine cases, mongoose cases, and human population density, as well as their within-site temporal deviations (change-from-baseline)^35^. Precipitation and temperatures were excluded from the treatment model because they reflect environmental conditions rather than observed behavior or sampling effort and distance to diagnostic facility was excluded because it is time-invariant. The outcome model (estimating mean observed bovine cases per district) used baseline shared-driver values (canine, mongoose, human population density) along with baseline precipitation, baseline temperature, and distance to diagnostic laboratory. Time-varying changes in precipitation and temperature were excluded from the outcome model to avoid absorbing the temporal trend of interest. The causal forest used the same six covariates as the outcome model to condition heterogeneity estimation. All three forests were fit using the *grf* package with num.trees = 2000 and num.threads = 4; all other parameters were left at package defaults and no additional hyperparameter tuning was performed. Nuisance model predictions were generated via the out-of-bag (OOB) mechanism native to the *grf* package, in which each tree is trained on a random subsample of observations and predictions are formed using only trees for which a given observation was not included in training^38^. Within each tree, an additional honesty constraint further partitions the subsample where half is used to determine splits and the other half to populate leaf estimates preventing overfitting without requiring explicit held-out folds^39^. The ATE was recovered using the same method discussed in the simulation section.

#### Hierarchical N-Mixture Model Application.

The N-mixture model framework provides yet another approach by explicitly separating the data-generating process into spillover and detection submodels. We adapted the hierarchical structure from^40^, where the biological submodel estimates true (unobserved) spillover occurrence with year as the predictor to estimate temporal trends, and includes random intercepts for district to account for spatial heterogeneity in baseline spillover risk. The detection submodel estimates the probability that a true spillover event is observed and reported using canine rabies cases, mongoose rabies cases, human population density, and distance to diagnostic laboratory as predictors. Because the N-mixture model estimates the biological process and detection process separately, we can include shared drivers of both the biological and detection processes, such as canine and mongoose cases, shared-drivers like canine and mongoose cases in the detection submodel without controlling away their causal effect on true bovine spillover in the biological submodel. Theoretically, this allows us to recover the temporal trend in true spillover risk while properly accounting for the influence of reservoir host dynamics on surveillance effort. This places the year coefficient on a different footing than in the GLMM and GAMM applications: because canine and mongoose cases are absent from the biological submodel, the year coefficient here captures the total effect of year on bovine spillover, inclusive of any indirect effect mediated through reservoir host dynamics. This estimand differs from GLMM and GAMM coefficients, which condition on canine cases in the count component, more closely approximate a direct effect of year. This estimand difference should be borne in mind when comparing trend magnitudes across methods.

We specified weakly informative priors for all parameters and fit the model using the *nimble* (*v1.3.0*; de Valpine *et al*. 2026) package with Markov chain Monte Carlo (MCMC) sampling. We ran 4 parallel chains for 500,000 iterations each, discarding the first 100,000 as burn-in and thinning by 200, yielding 2,000 retained samples per chain (8,000 total). The thinning interval was determined empirically from a pilot run by identifying the lag at which autocorrelation fell below 0.1 for all primary inferential parameters. Convergence was confirmed via the Gelman-Rubin diagnostic (R-hat ≤ 1.01 for all monitored parameters) and effective sample sizes were sufficient for all key parameters (ESS range: 4,562–7,477). We derived the temporal trend from the posterior distribution by exponentiating the year coefficient and converting it to percent change per year, providing an estimate on the same scale as the trends from the regression models. Like DML, this method is more methodologically complex than the GLMM and GAMM methods. As detailed in the simulation methods above, it could not be stabilized on the simulated data without strongly informative priors and was therefore excluded from the simulation benchmark.

We also tested this method as a candidate for the simulation study but excluded it after it failed to produce reliable trend estimates under weakly informative priors. See additional information in Supplementary Note 2.

## Supplementary Material

This is a list of supplementary files associated with this preprint. Click to download.

- GilbertsonNatCommsSupp.pdf

## Figures and Tables

**Figure 1:**
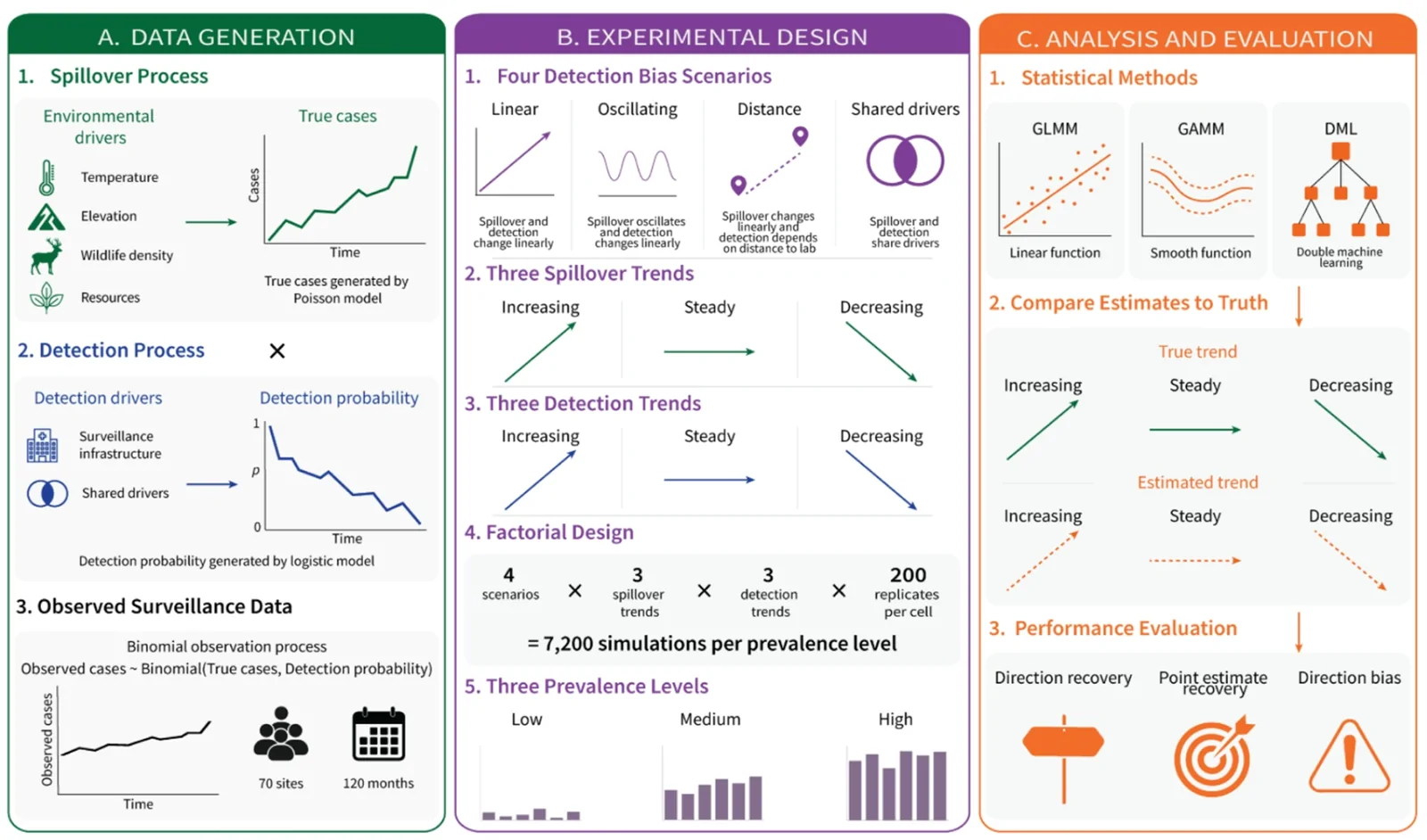
Overview of the simulation and analysis workflow. **(A)** True spillover cases are simulated from environmental drivers and detection probability is simulated separately from detection drivers using a logistic model. Observed surveillance data are generated by combining true cases and detection probability through a binomial observation process across 70 sites over 120 months. **(B)** Four scenarios represent detection bias structures, each crossed with three true spillover trends and three detection trends, repeated across three prevalence levels (low, medium, high). **(C)** Three statistical approaches (GLMM, GAMM, and DML) are fit to the observed data and their estimated trends are compared against the known true trend to assess direction recovery, point estimate recovery, and systematic direction bias.

**Figure 2:**
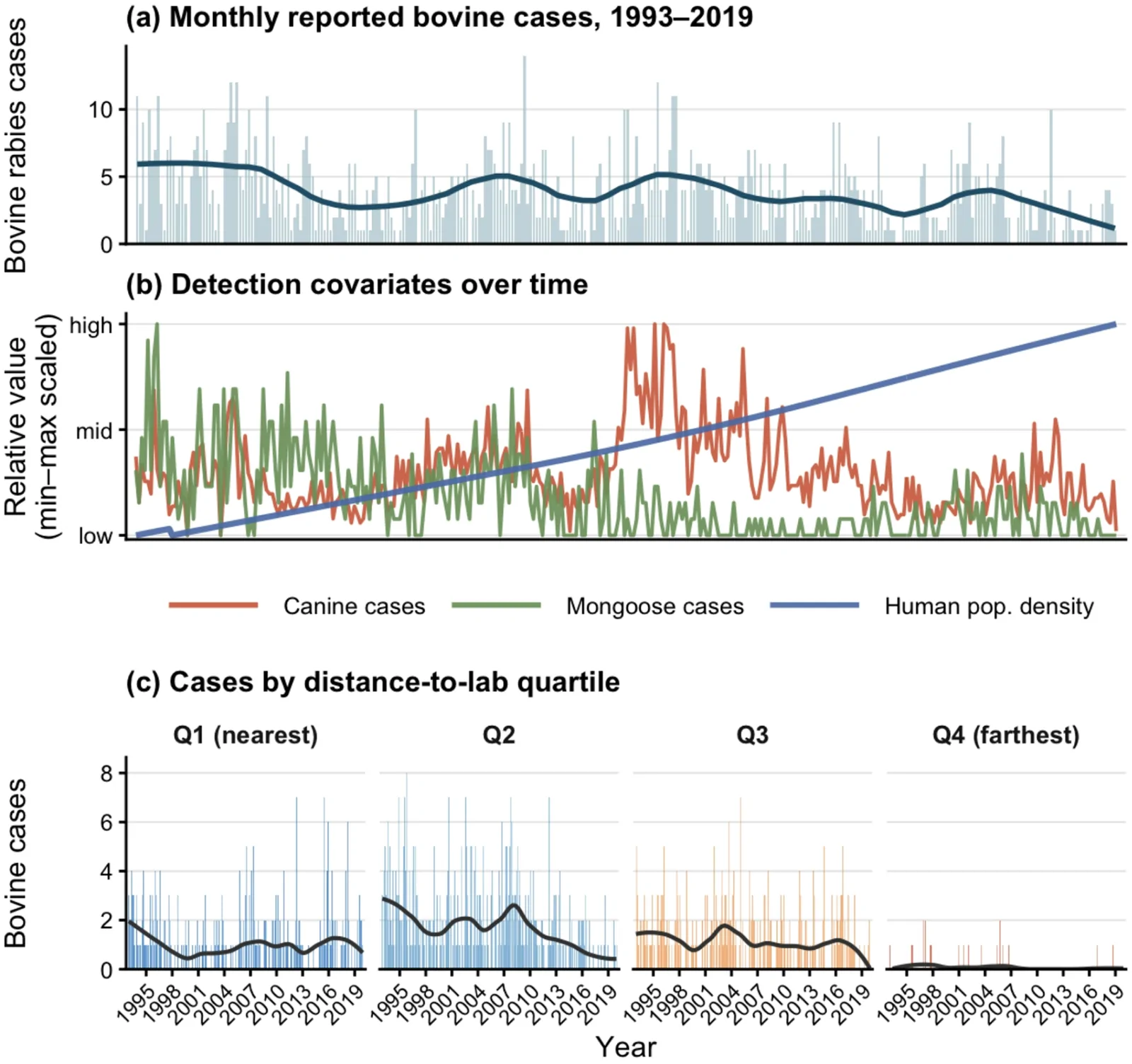
Raw bovine rabies surveillance data and detection covariates, 1993–2019. (a) Monthly reported bovine rabies cases summed across all districts, with loess smooth (span = 0.25). The apparent declining trend motivates investigation of whether changes in detection capacity contribute to the observed pattern. (b) Time-varying detection covariates averaged across districts and min–max scaled for visual comparability: canine rabies cases, mongoose rabies cases, and human population density. (c) Monthly bovine cases stratified by quartile of site-level distance to the diagnostic laboratory (Q1 = nearest, Q4 = farthest).

**Figure 3:**
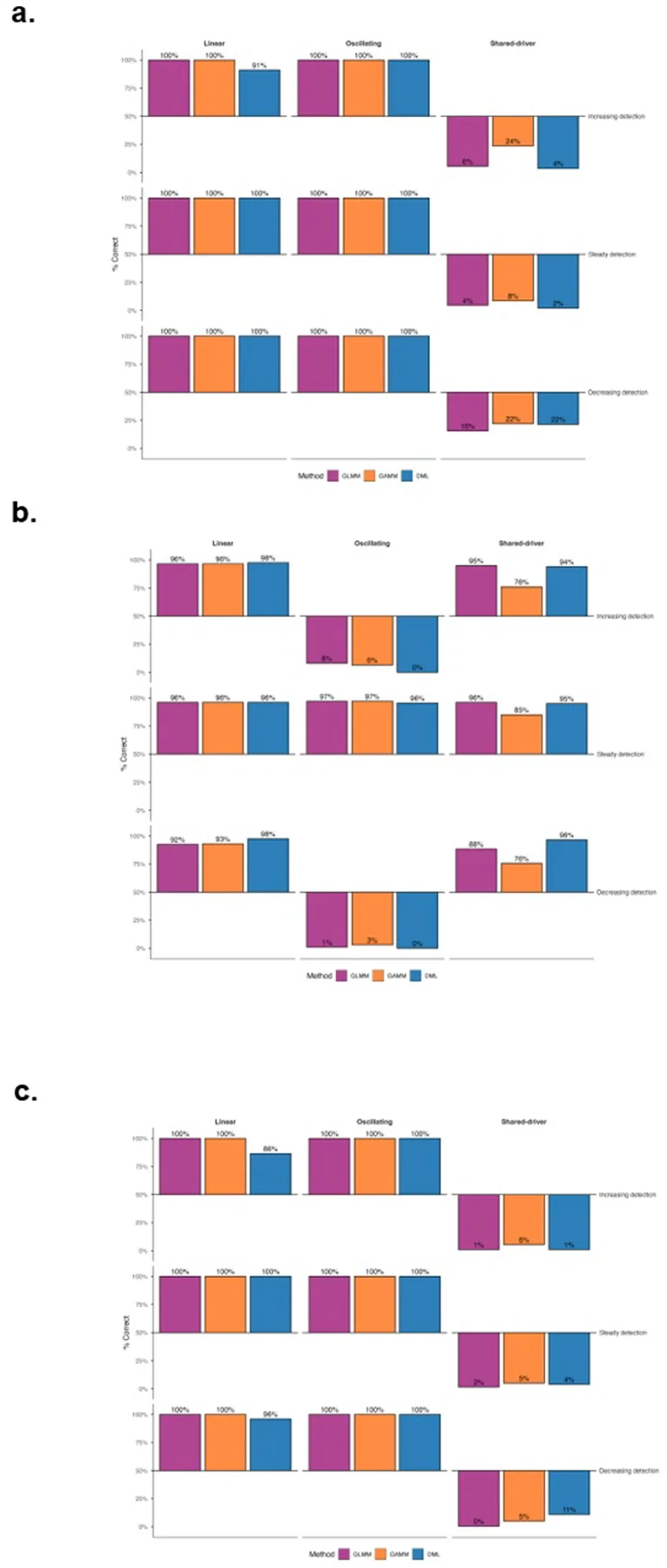
Direction recovery success for generalized linear mixed models (GLMMs), generalized additive mixed models (GAMMs), and double machine learning (DML) for linear, oscillating, and shared-driver scenarios at high prevalence. Panel a: increasing true spillover; b: steady; c: decreasing.

**Figure 4:**
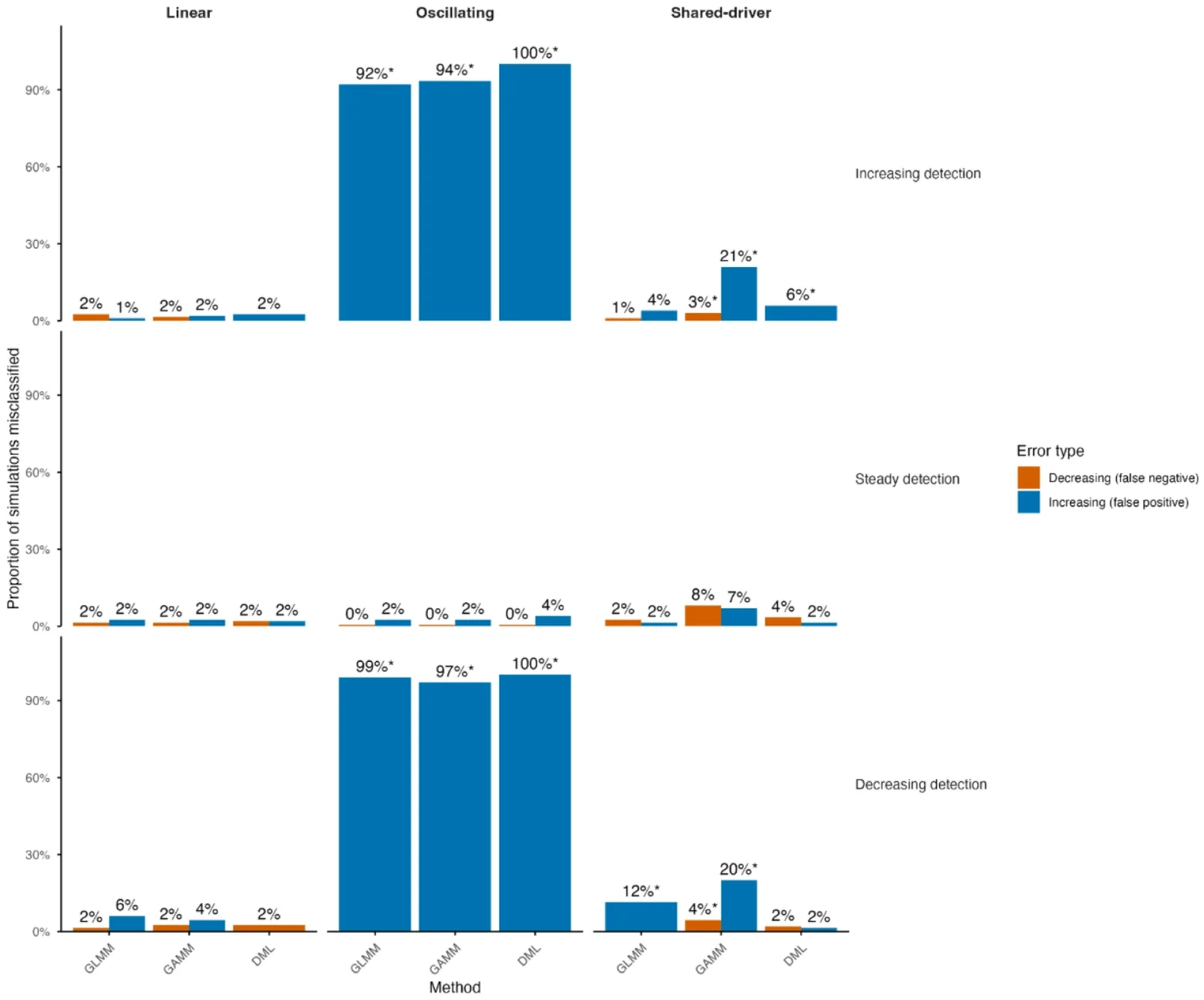
Misclassification errors for steady spillover trend in generalized linear mixed models (GLMMs), generalized additive mixed models (GAMMs), and double machine learning (DML) for linear, oscillating, and shared-driver scenarios at high prevalence. Denominator (n=200) is total simulations per scenario cell. * significant asymmetry between positive and negative errors (binomial test, Bonferroni-corrected p < 0.05, only tested when ten or more errors occurred for a given model).

**Figure 5:**
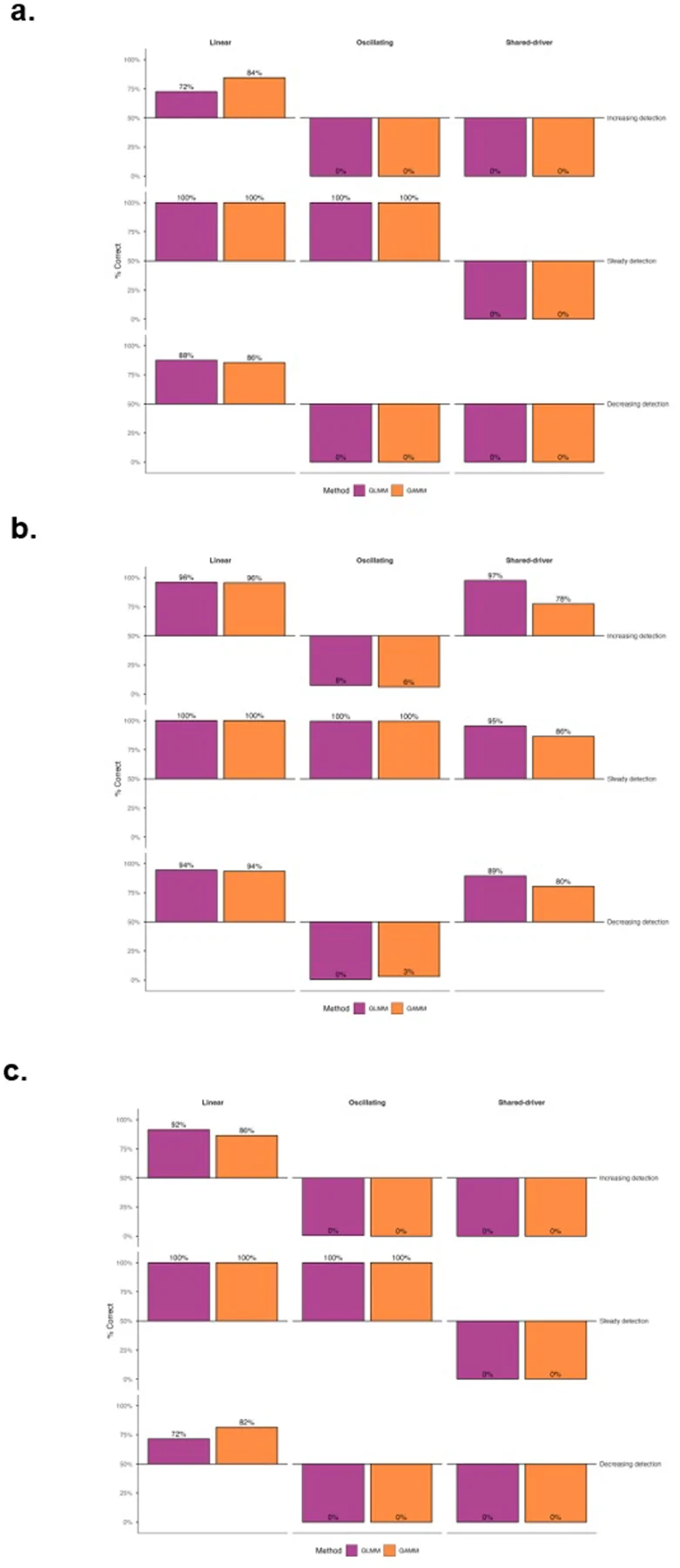
Point recovery success for generalized linear mixed models (GLMMs) and generalized additive mixed models (GAMMs) for linear, oscillating, and shared-driver scenarios. Panel a shows scenarios where the spillover is increasing over time, b is steady spillover over time, and c is decreasing spillover over time.

**Figure 6:**
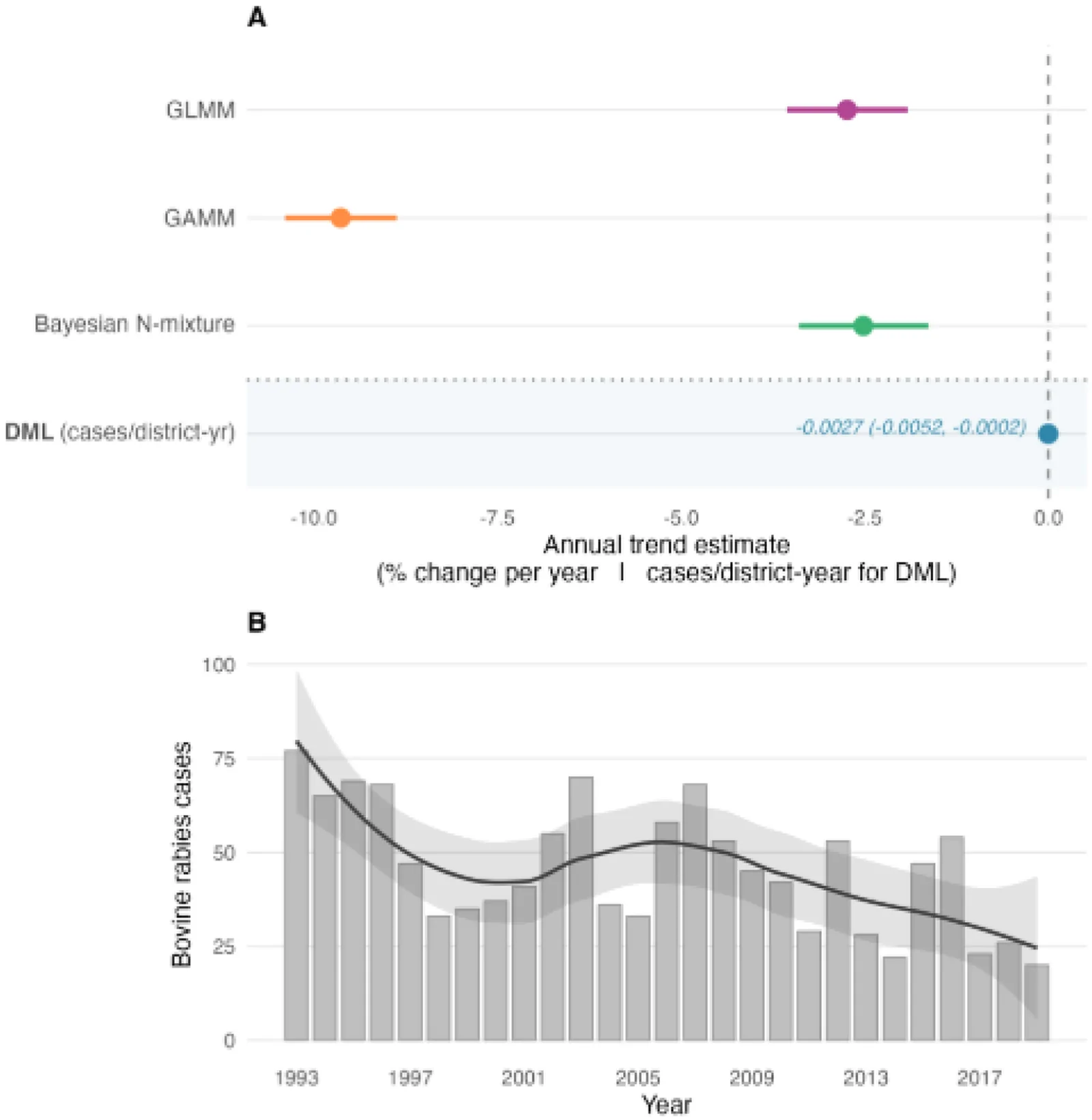
Recovered bovine rabies spillover trend over time for GLMM, GAMM, and Bayesian N-mixture models (panel A), and DML (panel B). Note that GLMM, GAMM, and hierarchical model results are percent change per year and DML estimates are the expected change in number of cases per year.

## Data Availability

The data will be available from the authors upon reasonable request.

## Box 1. Recommendations for interpreting and framing spillover trend analysis.

**Table:**

For analysts communicating results to decision-makers
**Report direction, not magnitude, as the headline finding.** Even when models recovered the correct trend direction, confidence intervals frequently failed to capture the true magnitude. Reserve point-estimate communication for simple, well-documented, low-bias systems (e.g., reportable high-consequence pathogens like human rabies). **Frame uncertainty as “should we act on this,” not “did we recover the truth.”** P(trend true | trend reported) is the question decision-makers actually face, and differs from P(trend recovered | true trend). DMĽs 55–71% reliability under shared-driver bias is a defensible basis for action *because that reliability is known* — imperfect methods remain useful once their failure rate is stated alongside the estimate. **Treat model disagreement as signal, not noise to average away.** In the rabies case study, GLMM, GAMM, and N-mixture models agreed on direction but differed in magnitude by more than threefold, driven by differences in count distribution and functional form. Report this spread rather than a single model's point estimate. **Flag oscillating/seasonal detection and low-prevalence settings as high-risk for false positives and false negatives, respectively.** Steady trends were most often misclassified as increasing under oscillating detection; at low prevalence, DML in particular can shrink toward falsely precise null estimates. Both failure modes should be disclosed, not just the point estimate. **Make surveillance assumptions explicit before modeling.** Use tools like DAGs, in consultation with subject-matter experts, to determine whether a dataset’s surveillance system plausibly resembles the shared-driver or oscillating-detection conditions under which all three methods failed.
For surveillance-system funders and public health agencies
**Treat surveillance investment as a methodological requirement, not only a resourcing preference.** The bias documented here is most pronounced exactly where infrastructure is weakest — regions also hypothesized to carry the highest spillover risk — so under-investment doesn’t just limit data volume, it systematically mislocalizes risk (inflating it where surveillance is improving, understating it where surveillance is contracting). **Where active surveillance isn’t feasible, collect effort-metadata alongside case counts.** Diagnostic testing volumes, healthcare-seeking indicators, and laboratory capacity changes over time allow analysts to distinguish real trends from detection artifacts — none of the adjustment strategies in prior literature (publication volume, GDP, press freedom, restricting to “low-bias” pathogens) captured the specific bias structures shown here to be most damaging. **Fund targeted validation studies and expert elicitation over one-time corrections.** Comparing detected cases against independent sampling, and structured elicitation of how detection probability has changed over time, are tractable near-term investments for existing passivesurveillance datasets that cannot be retrofitted with active surveillance. **Support sustained, longer-duration data collection.** Our simulations span a 10-year window; many influential rising-spillover claims span 3–4 decades. Whether these bias patterns compound or resolve over longer horizons is untested and directly relevant to the claims funders are being asked to act on.

## Code availability

The code is available from the authors upon reasonable request.
